# Comprehensive pan-cancer analysis and the regulatory mechanism of AURKA, a gene associated with prognosis of ferroptosis of adrenal cortical carcinoma in the tumor micro-environment

**DOI:** 10.3389/fgene.2022.996180

**Published:** 2023-01-04

**Authors:** Keqiang Lu, Xingxing Yuan, Lingling Zhao, Bingyu Wang, Yali Zhang

**Affiliations:** Department of Gastroenterology, Heilongjiang Academy of Traditional Chinese Medicine, Harbin, China

**Keywords:** AURKA, pan-cancer analysis, tumor micro-environment, regulatory mechanism, ferroptosis

## Abstract

**Background:** The only curative option for patients with locally or locally advanced adrenocortical carcinoma is primary tumor curative sexual resection (ACC). However, overall survival remains low, with most deaths occurring within the first 2 years following surgery. The 5-year survival rate after surgery is less than 30%. As a result, more accurate prognosis-related predictive biomarkers must be investigated urgently to detect patients’ disease status after surgery.

**Methods:** Data from FerrDb were obtained to identify ferroptosis-related genes, and ACC gene expression profiles were collected from the GEO database to find differentially expressed ACC ferroptosis-related genes using differential expression analysis. The DEFGs were subjected to Gene Ontology gene enrichment analysis and KEGG signaling pathway enrichment analysis. PPI network building and predictive analysis were used to filter core genes. The expression of critical genes in ACC pathological stage and pan-cancer was then investigated. In recent years, immune-related factors, DNA repair genes, and methyltransferase genes have been employed in diagnosing and prognosis of different malignancies. Cancer cells are mutated due to DNA repair genes, and highly expressed DNA repair genes promote cancer. Dysregulation of methyltransferase genes and Immune-related factors, which are shown to be significantly expressed in numerous malignancies, also plays a crucial role in cancer. As a result, we investigated the relationship of AURKA with immunological checkpoints, DNA repair genes, and methyltransferases in pan-cancer.

**Result:** The DEGs found in the GEO database were crossed with ferroptosis-related genes, yielding 42 differentially expressed ferroptosis-related genes. Six of these 42 genes, particularly AURKA, are linked to the prognosis of ACC. AURKA expression was significantly correlated with poor prognosis in patients with multiple cancers, and there was a significant positive correlation with Th2 cells. Furthermore, AURKA expression was positively associated with tumor immune infiltration in Lung adenocarcinoma (LUAD), Liver hepatocellular carcinoma (LIHC), Sarcoma (SARC), Esophageal carcinoma (ESCA), and Stomach adenocarcinoma (STAD), but negatively correlated with the immune score, matrix score, and calculated score in these tumors. Further investigation into the relationship between AURKA expression and immune examination gene expression revealed that AURKA could control the tumor-resistant pattern in most tumors by regulating the expression level of specific immune examination genes.

**Conclusion:** AURKA may be an independent prognostic marker for predicting ACC patient prognosis. AURKA may play an essential role in the tumor microenvironment and tumor immunity, according to a pan-cancer analysis, and it has the potential to be a predictive biomarker for multiple cancers.

## Introduction

Adrenal cortical carcinoma (ACC) is a rare malignant tumor with an annual incidence of one to two per million that can occur at any age and is more common in women (Cheng et al., 2021; Faron et al., 2022; Pitsava et al., 2022). It is an incidental adrenal tumor and one of the most common reasons for adrenalectomy, accounting for 14% of all spontaneous adrenal tumors (Alyateem and Nilubol, 2021). Although radical resection is the only option for the majority of ACC patients, postoperative survival remains low. As a result, understanding the molecular mechanism of ACC and identifying key target molecules can help predict tumor prognosis.

Currently, ACC is diagnosed using hormone detection and imaging, which plays a vital role in the initial diagnosis and prognostic detection and necessitates repeated detection (Mete et al., 2022). Efforts have been made for decades to discover new reliable, usable diagnostic and prognostic factors. Despite these achievements, 5-year mortality remains higher than 50% (Mizdrak et al., 2021). Accordingly, it is critical to discover new biomarkers that can predict patient outcomes and provide new treatment options.

Ferroptosis, a distinct mechanism of cell death caused by iron-dependent phospholipid peroxidation, has been shown to damage treatment-resistant cancer cells, particularly those in mesenchymal condition and prone to metastasis (Jiang et al., 2021). Correlative research has demonstrated that ferroptosis-related genes are linked to prognosis in various malignancies, including uveal melanoma, glioma, and adrenocortical tumors (Chen et al., 2021a; Luo and Ma, 2021; Zheng et al., 2021).

Aurora kinase A (AURKA) is a serine/threonine kinase family member, and its activation has been linked to several malignancies. Several studies have shown that highly expressed AURKA can be used as a prognostic marker in various malignancies, including ACC (Du et al., 2021; Tang et al., 2021; Zhang et al., 2022).

Tumor samples from GEO databases were combined with standard models in this study. Differential expression analysis and ACC predictive analysis revealed significantly correlated genes. Pan-cancer analysis was used to study the expression of target genes in 40 different types of cancer. Then the correlations between target gene expression and tumor immune microenvironment, immune checkpoints, DNA repair genes, and methyltransferase were discovered.

## Materials and methods

### Data source

The GEO database (https://www.ncbi.nlm.nih.gov/geo) was used to download the RNA expression data for ACC from accession numbers GSE12368, GSE19750, and GSE75415, which contained 17 regular and 74 tumor tissues. All data were quantile normalized using a log2-scale transformation. The gene symbols found in multiple probes were calculated using their mean expression levels.

### Ferroptosis-related genes

The “Limma” package of R software was used to investigate the differential expression genes (DEGs) of ACC (version: 3.42.2). *p*-values were adjusted to account for false-positive results. The number of highly expressed molecules in groups 1 (tumor) and 2 (standard control) that met the |log2(FC)|>1&p. Adj0.05 threshold was counted. The DEGs were also visualized using the “ComplexHeatmap” and “ggplot2” packages. The DEGs and ferroptosis-related genes were then intersected to obtain ferroptosis-related genes with differential expression (DEFGs).

### Functional analysis

Metascape Online (https://metascape.org/gp/index.html#/main/step1) was used for available analysis. Metascape was used to perform functional analysis and build a PPI network using the ferroptosis-related genes. MCODE was used to reveal more densely connected regions.

### Construction and prognostic value of IRSS

Univariate (Wei et al., 2022) Cox regression model is a semi-parametric regression model. The model’s dependent variables are survival results and survival time. It may examine the impact of several variables on survival time simultaneously. It does not require estimated data and can evaluate data with suppressed survival time. The least absolute shrinkage and selection operator (LASSO) is an L1-regularized linear regression approach. Using L1-regularization, part of the learned feature weights will be set to zero, achieving the goal of sparsity and feature selection (Tian et al., 2022). Univariate Cox regression analysis of DEFGs was used to identify significant prognosis-related genes, followed by LASSO regression analysis to obtain independent genes. A multivariate Cox regression analysis was also performed to obtain regression coefficients for independent prognostic factors. Finally, an immune risk score signature (IRSS) based on the Cox regression coefficient beta value was developed.

### Survival analysis

One-way Cox was used to analyze the association of ACC expression with patient survival, and Xian Tao Academic created a forest plot of the correlation of overall survival and disease-specific survival of ARUKA in pan-cancer (https://www.xiantao.love).

### Immune correlation analysis

The TIMER database was used to download data from multiple immune-infiltrating cells in 40 cancers, and the correlation between target gene expression and immune cell scores was examined separately. A lollipop graph of the correlation of target genes with immune cells in the cancer microenvironment and a diagram of the correlation of target genes with immune scores, stromal scores, and computational scores in five cancers were drawn using Xian Tao Academic (https://www.xiantao.love).

### Correlation analysis of DNA repair genes and methyltransferases

Using the TCGA expression profiling data, the correlation of DNA repair genes with target gene expression was assessed. The relationship between methyltransferases and the target gene was also investigated. Xian Tao Academic (https://www.xiantao.love) was used to create heat maps, with red dots indicating significant correlations.

## Results

### Results of DEGs screening in ACC

The information on the GEO database used is listed in Table 1. A total of 2,311 differentially expressed genes were identified following differential gene analysis: in GSE12368, the total number of molecules after filtering was 21,655, of which 849 IDs met the |log2(FC)|>1&p. Adj0.05 threshold. There were 170 highly expressed (logFC is positive) individuals in the standard group and 679 highly expressed (logFC is negative) individuals in the tumor group. The number of molecules in GSE19750 after filtering is 21,655, and 849 IDs meet the |log2(FC)|>1&p. Adj0.05 threshold.

**TABLE 1:** The information of datasets from the GEO database.

Accession number	Platform	Samples	Experiment type
GSE75415	GPL96	25	expression profiling by array
GSE12368	GPL570	18	expression profiling by array
GSE19750	GPL570	48	expression profiling by array

**FIGURE 1:**
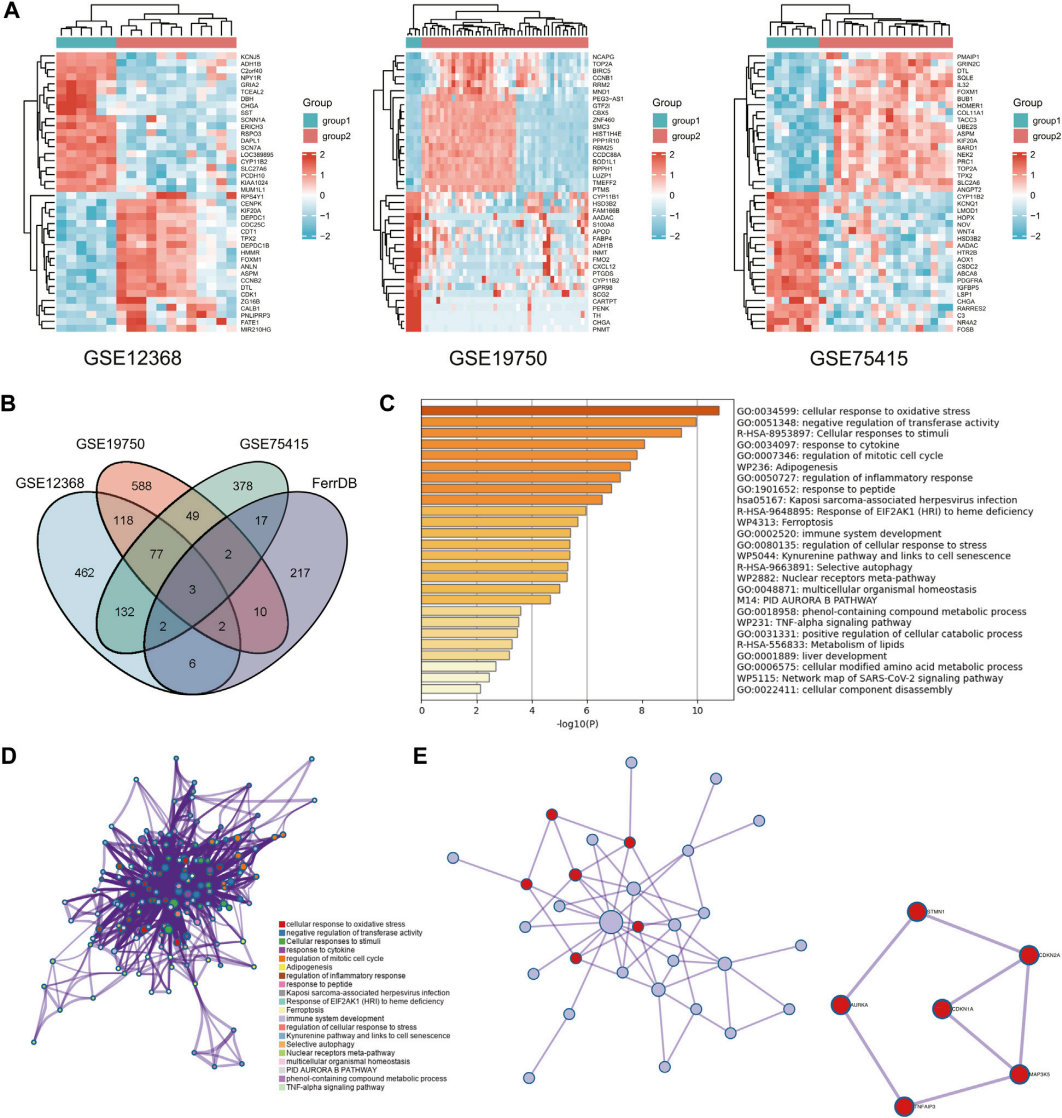
Access to key genes. **(A)** Heatmap of differentially expressed genes in three GEO databases. **(B)** Venn diagram of differential ferroptosis genes. **(C,D)** Graph showing the GO and KEGG analysis based on the Metascape Online, bar plot, and network showing the distribution and relationship of the different functions. **(E)** PPI network and MCODE reveal hub genes in differential ferroptosis gene sets.

Under this threshold, the regular group has a high expression (logFC is positive). The number was 170, with 679 having a high face (logFC is negative) in the tumor group. The number of molecules in GSE19750 after filtering is 21,655, and 849 IDs meet the |log2(FC)|>1&p. Adj0.05 threshold. Under this threshold, the usual group has a high expression (logFC is positive). The number was 170, with 679 highly expressed (logFC is negative) in the tumor group. After filtering in GSE75415, 12,548 molecules were obtained, of which 660 dysregulated genes satisfy |log2(FC)|>1&p. Adj0.05; under this threshold, the number of highly expressed (logFC is positive) genes in the standard group is equal to the number of highly expressed (logFC is positive) genes in the standard group. There were 258 in the tumor group, with 402 being highly expressed (logFC is negative) (Figure 1A). DEFGs was created by intersecting DEGs from GEO databases and ferroptosis-related genes (Figure 1B).

We used Metascape Online to perform a functional analysis to investigate ACC’s underlying mechanisms of ferroptosis signatures. The Gene Ontology (GO) analysis results show that these DEFGs were primarily enriched in response to stimuli, oxidative stress responses, immune system processes, and negative regulators of transferase activity, as shown in Figures 1C, D. According to the Kyoto Encyclopedia of Genes and Genomes (KEGG) pathway analysis, these d DEFGs were primarily enriched in ferroptosis, cellular responses to stimuli, selective autophagy, and EIE2AKI response to heme deficiency. As a result of these findings, we decided to investigate the relationship between the ferroptosis-gene set and the tumor immune microenvironment. Furthermore, the MCODE plugin and the MetascapeOnline-based protein-protein interaction (PPI) network identified necessary modules in these filiform genes (Figure 1D). STMN1, CDKN2A, CDKN1A, MAP3K5, TNFAIP3, and AURKA are involved in seven edges and six nodes.

### Construction and prognostic value of IRSS

**FIGURE 2:**
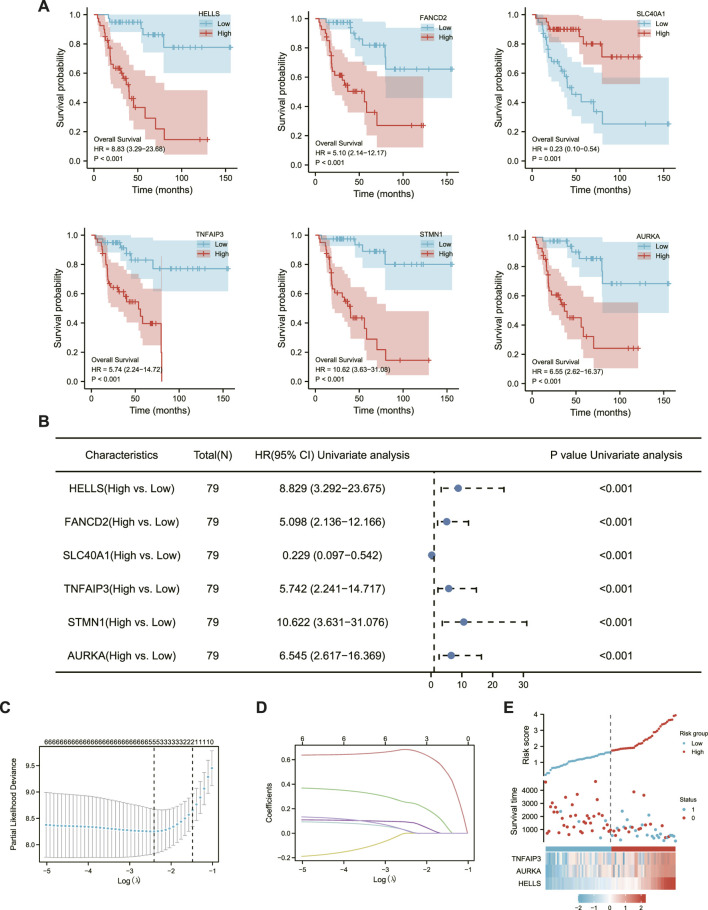
Establishment of ACC ferroptosis-related prognostic model. **(A)** six Significantly Differential Gene Survival Analysis Survival Chart. **(B)** Forest plot showing the results of a univariate Cox regression analysis. **(C)** Ten-fold cross-validation plot. **(D)** LASSO coefficient trajectory diagram. **(E)** The risk score, survival status and heat map of three key genes in patients.

The associations of 42 DEFGs with overall survival in ACC were calculated separately using univariate survival analysis. Six genes were significantly related to ACC prognosis, including AURKA, TNFAIP, HELLS, STMN1, FANCD2, and SLC4OA1. The high expression of the six genes associated with poor prognosis in ACC, as shown in Figure 2A, greatly impacted the overall survival of ACC patients and was followed by LASSO regression analysis.

LASSO regression can improve model accuracy and interpretability while also eliminating the issue of collinearity between independent variables (Yu et al., 2021). The results of Figures 2C, D determined that the model fit best when the penalty coefficient was 3, and the corresponding three immune genes, TNFAIP3, AURKA, and HELLS, were included in the model (Figure 2E).

**FIGURE 3:**
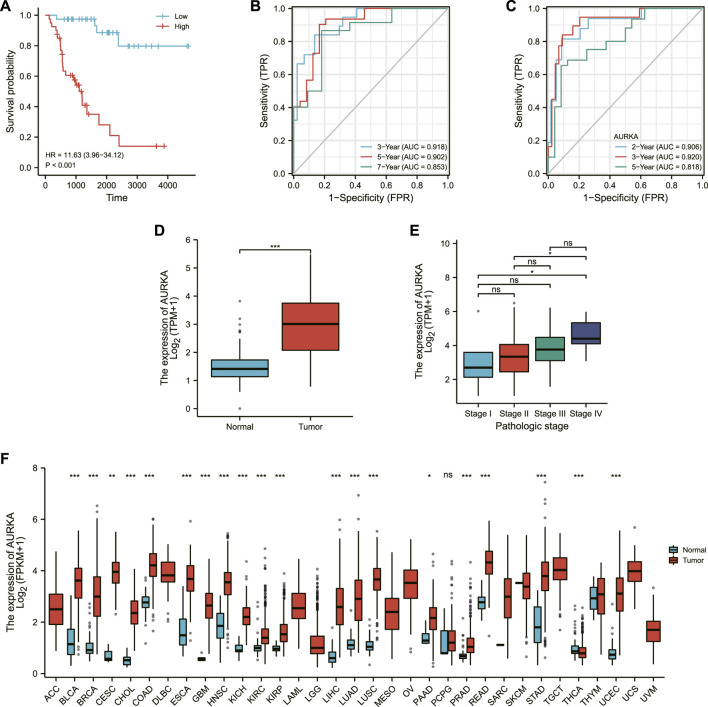
Validation of the model. **(A)** Survival map of high and low risk patients. **(B)** 3-Gene time-dependent ROC plot. **(C)** Single-gene time-dependent ROC plot. **(D)** Expression of AURKA in normal population and ACC patients. **(E)** Expression of AURKA in ACC patients at different stages. **(F)** AURKA expression in a wide range of cancers.

Each patient’s risk score was calculated as previously described (Peng et al., 2022). Furthermore, the risk score of each ACC patient was directly computed using the above formula. The samples were then divided into high- and low-risk groups, which were then grouped based on the median. The KM curve results showed that the high-risk group had a worse prognosis than the low-risk group (Figure 3A, log-rank *p* 0.001; HR = 11.63% CI = 3.9634,12).

The area under the receiver operating characteristic (ROC) curve (AUC) was used to assess IRSS’s prognostic predictive value in ACC patients. The receiver operating characteristic curves are referred to as ROC curves, with sensitivity as the ordinate and 1-specificity as the abscissa (DeLong et al., 1988). The AUC is a probability value ranging from 0.5 to -1 that is used to evaluate the accuracy of the model prediction; a more extensive area indicates higher accuracy. In the current study, the greater its value, the greater the agreement between predicted and actual overall survival.

The area under the curve (AUC) was 0.918 (3-year OS), 0.902 (5-year OS), and 0.853 (7-year OS), as shown in Figure 3B, indicating that the prediction model was well established. We also created ROC curves for the effect of AURKA alone on survival time in ACC patients, with AUCs of 0.906 (2-year OS), 0.920 (3-year OS), and 0.818 (5-year OS) (Figure 3C). The above results demonstrated the model’s robustness and accuracy in predicting patient prognosis. Simultaneously, we discovered that AURKA’s single-gene and polygenic prognostic models have similar prediction results. AURKA is a common intersection of ferroptosis-related genes and three differentially expressed gene sets in the GEO database. As a result, we make the bold assumption that AURKA is a crucial gene associated with ferroptosis prognosis in ACC. Then, we looked at AURKA’s pan-cancer expression and its relationship to ACC pathological stage.

### Expression of AURKA in pan-cancer

The expression level of AURKA was higher in ACC tissue (Figure 3D), and the expression level of AURKA in different stages of ACC was shown in Figure 3E, indicating that the expression level of AURK increased with the progression of ACC. We then investigated AURKA expression in pan-cancer, and the results show that AURKA was highly expressed in all 31 tumors except PCPG and THCA (Figure 3F).

### Prognostic analysis of AURKA expression in ACC and other cancers

**FIGURE 4:**
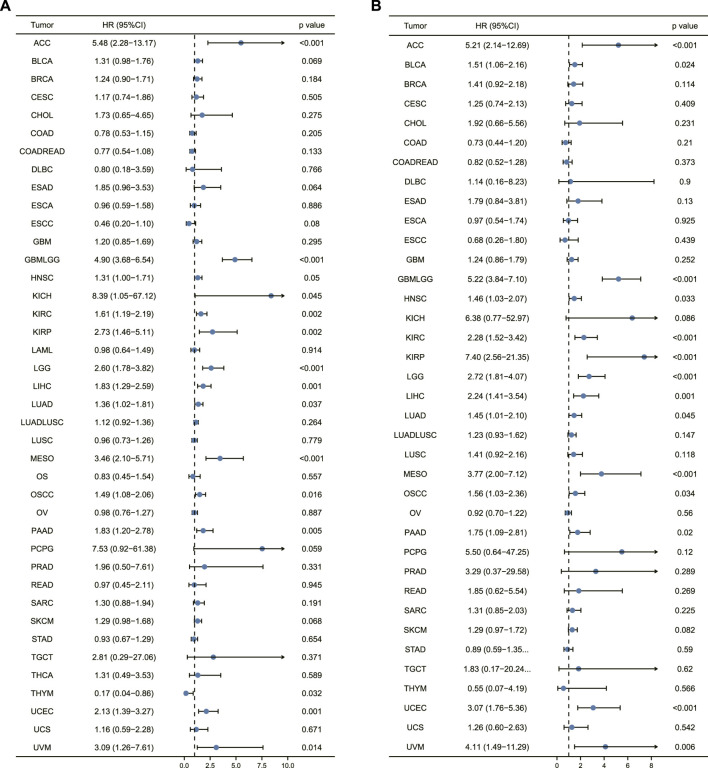
Prognostic analysis of AURKA in pan-cancer. **(A)** Forest plot of overall survival prognostic analysis of AURKA in pan-cancer. **(B)** Disease-specific survival prognostic analysis of AURKA in pan-cancer.

The correlation of AURKA expression with overall survival and disease-specific survival in 40 TCGA tumors was calculated using univariate survival analysis. AURKA expression, as shown in Figure 4A, significantly impacted overall survival in multiple cancers.

In addition to COAD, COADREAD, DLBC, ESCC, and THYM, forest plot results revealed that high AURKA expression was associated with poor patient prognosis. Figure 4B depicts the correlation of AURKA expression with disease-specific survival, demonstrating that in ACC, GBMLGG, KICH, KIRC, KIRP, and LGG, patients with high AURKA expression had significantly lower disease-specific survival than patients with common AURKA expression. Overall, the findings suggest that AURKA could be used to predict the prognosis of ACC and other cancers.

### Correlation of AURKA with immune cells in the pan-cancer microenvironment

**FIGURE 5:**
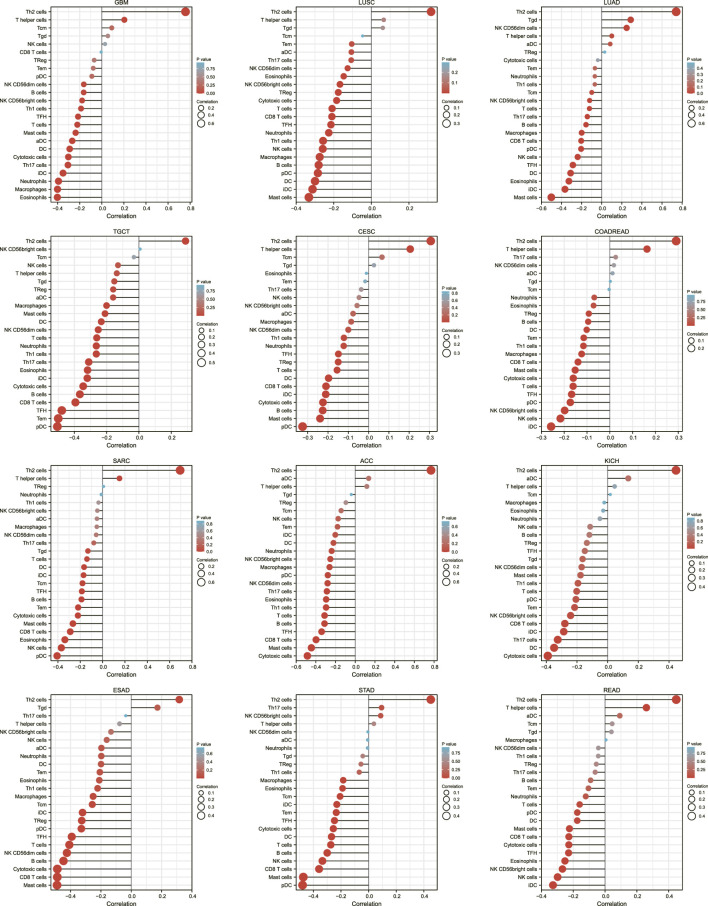
Lollipop plot of AURKA’s association with immune cells in 12 cancers.

It has been studied whether AURKA expression correlates with immune infiltration in ACC or other types of cancer. The findings revealed that AURKA expression is associated with the level of immune infiltration in various tumors. Particularly Th2 cells. AURKA was significantly positively correlated with Th2 cells in all 40 cancers studied, and it was the first positive correlation. We also chose 12 cancers to map the relationship between AURKA and immune cells in these cancer microenvironments (GBM, LUSC, LUAD, TGCT, CESC, COADREAD, SARC, ACC, KICH, ESAD, STAD, READ). Figure 5 shows that, in addition to Th2 cells, many other immune cells were negatively correlated with AURKA. The killer CD8+T regulated by Th1 was the main focus of the previous immunotherapy study for AURKA. Perhaps Th1-executing B Cells will have an unanticipated effect on AURKA targeted therapy. AURKA may also inhibit other immune cells in the tumor microenvironment, though the specific mechanism is unknown.

**FIGURE 6:**
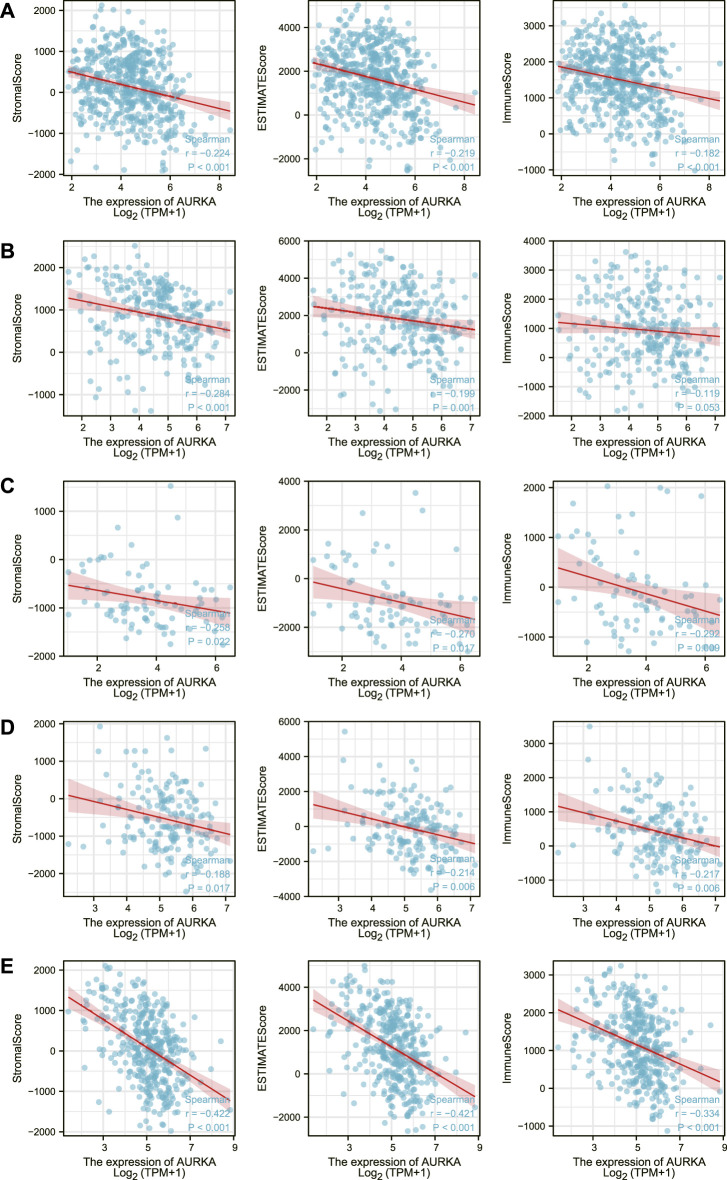
Correlation of AURKA expression level with immune score, stroma score, calculated score in LUAD **(A)**, SARC **(B)**, ACC **(C)**, ESCA **(D)**, STAD **(E)**.

Xiantao Academic then created a correlation chart of AURKA expression levels in LUAD, SARC, ACC, ESCA, STAD, immune score, matrix score, and calculation score, which were all negatively correlated (Figure 6).

### Correlation of AURKA expression with immune checkpoints

**FIGURE 7:**
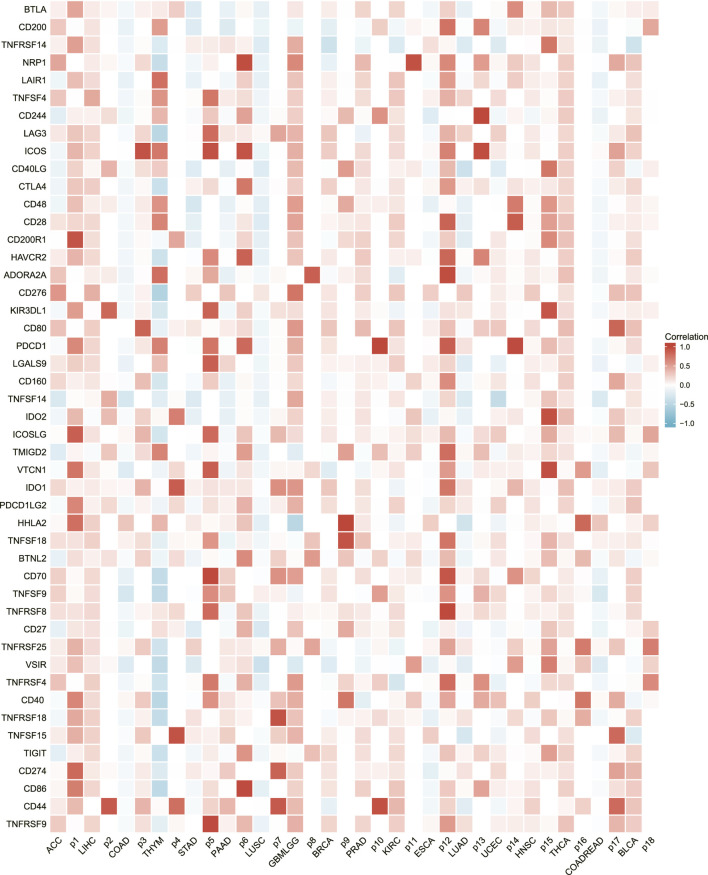
Heatmap of AURKA’s association with immune checkpoints in a broad range of cancers.

More than 40 common immune checkpoint genes were analyzed, as was the relationship between AURKA expression and immune checkpoint gene expression. Figure 7 depicts the results. AURKA was positively correlated with the presentation of immune checkpoint genes in many cancers, which supports our findings in Figure 5. Meanwhile, we discovered that AURKA was significantly negatively associated with most checkpoint genes in thymic carcinoma. The thymus is the site of T cell maturation and a mechanism that inhibits the AURKA-mediated increase in immune checkpoint expression, protecting T cells in the thymus.

### The relationship between AURKA expression and DNA repair gene and methyltransferase expression

**FIGURE 8:**
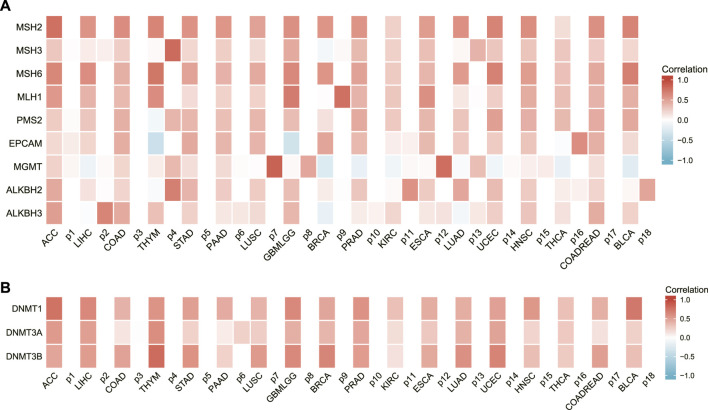
The relationship between AURKA expression and DNA repair gene and methyltransferase expression. **(A)** Heatmap of correlations between AURKA and DNA repair genes. **(B)** Heatmap of correlation between AURKA and methyltransferase genes.

AURKA was found to be associated with DNA repair genes as well as methyltransferase genes in several common cancers, as shown in Figures 8A, B. AURKA may have an indirect effect on cancer development and progression by modulating epigenetic status.

## Discussion

Although the incidence of adrenal cortical carcinoma is very low, it is one of the most aggressive solid tumors with a poor prognosis (Yeoh et al., 2022). Furthermore, the recurrence of ACC patients after surgery is still common. As a result, more biomarkers are required for more accurate predictive detection in ACC patients to improve the detection of postoperative risk. The discovery of predictive cancer biomarkers can aid in predicting each patient’s prognosis (Mizdrak et al., 2021; Lippert et al., 2022; Waszut and Taylor, 2022). Using robust rank analysis and a PPI network, XiaoH et al. identified five genes (TOP2A, NDC80, CEP55, CDKN3, and CDK1) that could predict the prognosis of ACC (Xiao et al., 2018). Giordano et al.‘s laid the groundwork for ACC molecular classification and prediction, as well as a rich source of potential diagnostic and prognostic markers (Xu et al., 2019).

Ferroptosis is a new iron-dependent programmed cell death method discovered that can induce cell death by promoting cellular lipid peroxidation. It is involved in the occurrence and development of many diseases and plays an essential regulatory role in disease processes. Related studies have shown that ferroptosis plays a role in the progression of various cancers. For example, inhibiting glutathione synthesis in ccRCC in clear cell renal cell carcinoma can induce ferroptosis and inhibit tumor growth (Miess et al., 2018); According to other research (Liu et al., 2020; Chen et al., 2021b; Lu et al., 2022), ferroptosis attenuates the viability of glioma cells, and activation of ferroptosis inhibits glioma cell proliferation. Inhibition of ferroptosis accelerates glioma proliferation and metastasis and promotes angiogenesis and malignant transformation of gliomas. One study discovered that ferroptosis sensitivity was significantly increased in adrenocortical carcinoma and proposed ferroptosis induction as a treatment option for ACC (Belavgeni et al., 2019).

We obtained six critical genes in this study by crossing the up-regulated genes in ACC with the genes associated with overall survival in ACC. Three of them were chosen to build a polygenic model. The AURKA prediction model and the polygenic model produced very similar results. Meanwhile, AURKA is the point of convergence for the ferroptosis-related gene set and the GEO databases. As a result, we boldly identified AURKA as a critical gene in ACC ferroptosis. We then looked at AURKA expression in ACC and other cancers to see if it had any predictive value. The findings revealed that AURKA was highly expressed in ACC and most cancers and that its expression level increased as ACC progressed. It is consistent with previous research findings (Naso et al., 2021; Sankhe et al., 2021; Ng et al., 2022; Wang et al., 2022). Related studies have also shown high levels of AURKA as an indicator of poor prognosis in bladder cancer. It is also associated with the development and prognosis of rectal cancer, hepatocellular carcinoma, and head and neck cancer (Lu et al., 2021; Tsepenko et al., 2021; Guo et al., 2022; Huang et al., 2022). This research discovered that high AURKA was related to a bad prognosis in various malignancies by creating a deep forest graph and feeding back the association between AURKA, overall survival, and disease-specific survival. It gives compelling evidence that ARUKA may be used to predict the prognosis of ACC and other malignancies.

In addition, we investigated the relationship between AURKA and immune cells in the pan-cancer microenvironment. We discovered that AURKA had a substantial positive link with Th2 cells in all 40 malignancies studied, and these were all the first positive correlations. We next chose 12 malignancies to investigate the association between AURKA and immune cells in them, finding that all immune cells except Th2 cells were adversely connected with AURKA. Previous research (Bustos-Moran et al., 2019; Sun et al., 2021; Long and Zhang, 2022) has shown that AURKA may impact T cells, reshape the immunosuppressive tumor microenvironment, apoptosis, and hypoxia and hence contribute to immunological control, particularly CD8^+^ T cells that govern Th1 regulation. For example, studies (Han et al., 2020) suggest that decreasing Aurora-A activity or deleting the AURKA gene might boost IL10-induced infiltration and growth of CD8^+^ T cells in malignancies. Th1-executing B Cells may have unanticipated impacts on AURKA targeted treatment. AURKA may also block other immune cells in the tumor microenvironment, albeit the particular mechanism is unknown. We next looked at the relationship between AURKA expression level and immunological score, stromal score, and computational score in five malignancies (ACC, SARC, LUAD, ESCA, and STAD), which were all shown to be negatively linked. AURKA has also been identified to affect tumor immunological patterns in diverse malignancies by controlling the expression of particular immune checkpoint genes, according to subsequent research. AURKA was shown to be favorably connected with the indication of immune checkpoint genes, which supports our prior results from a pan-cancer immunological correlation study. The discovery of immunological checkpoints opens up new avenues for tumor therapy. Immune checkpoint inhibitors have been employed in treating many tumors recently, and their effectiveness and safety have been objectively validated (Cai et al., 2022; Minegishi et al., 2022). In addition, we discovered an intriguing phenomenon. AURKA was strongly inversely related to most checkpoint genes in thymic cancer. The thymus is the location of T cell maturation. Thymic cancer has a mechanism that blocks the AURKA-mediated rise in immune checkpoint expression, safeguarding T cells in the thymus.

DNA repair capacity, which is primarily determined by repair gene expression levels, is the first line of defense against genotoxic stress, which causes metabolic changes, inflammation, and cancer, and is also required for maintaining genome stability and protecting cells from endogenous and exogenous DNA traumatic injuring (Shao et al., 2022; Zuo et al., 2022).

This study looked at nine DNA repair genes: MSH2, MSH3, MSH6, MLH1, PMS2, EPCAM, MGMT, ALKBH2, and ALKBH3. In most malignancies, AURKA expression was strongly positively linked with DNA repair genes, according to the findings. Furthermore, the results of this study revealed that the levels of ARUKA and the methyltransferase gene expression exhibited a substantial positive link in a range of malignancies.

## Conclusion

To summarize, we did differential expression analysis on the GEO database data, obtaining DEFGs by intersecting with ferroptosis-related genes and exploring some information from them. The significant result is that AURKA is a critical gene for the prognosis of ferroptosis in ACC and can be exploited as an ACC biomarker. The expression of ARUKA is connected with the tumor microenvironment and the number of immune cells in the pan-cancer study, which can impact cancer growth by controlling the level of immune cells, DNA repair, and DNA methylation. This result can only be reached from bioinformatics research, and thus further biological tests are required to demonstrate ARUKA’s probable relevant activities, action mechanisms, and signaling pathways in ACC ferroptosis. It is believed that this work would aid in related research while providing additional biological information about the mechanism of AURKA in tumor immunity and the tumor microenvironment in future research.

## Data Availability

The original contributions presented in the study are included in the article/Supplementary Materials, further inquiries can be directed to the corresponding author.

## References

[B1] Alyateem G. Nilubol N. (2021). Current status and future targeted therapy in adrenocortical cancer. Front. Endocrinol. 12, 613248. 10.3389/fendo.2021.613248 PMC7957049 33732213

[B2] Belavgeni A. Bornstein S. R. von Massenhausen A. Tonnus W. Stumpf J. Meyer C. (2019). Exquisite sensitivity of adrenocortical carcinomas to induction of ferroptosis. Proc. Natl. Acad. Sci. U. S. A. 116, 22269–22274. 10.1073/pnas.1912700116 31611400 PMC6825277

[B3] Bustos-Moran E. Blas-Rus N. Alcaraz-Serna A. Iborra S. Gonzalez-Martinez J. Malumbres M. (2019). Aurora A controls CD8(+) T cell cytotoxic activity and antiviral response. Sci. Rep. 9, 2211. 10.1038/s41598-019-38647-y 30778113 PMC6379542

[B4] Cai Z. Zhan P. Song Y. Liu H. Lv T. (2022). Safety and efficacy of retreatment with immune checkpoint inhibitors in non-small cell lung cancer: A systematic review and meta-analysis. Transl. Lung Cancer Res. 11, 1555–1566. 10.21037/tlcr-22-140 36090645 PMC9459604

[B5] Chen Q. Wang W. Wu Z. Chen S. Chen X. Zhuang S. (2021). Over-expression of lncRNA TMEM161B-AS1 promotes the malignant biological behavior of glioma cells and the resistance to temozolomide via up-regulating the expression of multiple ferroptosis-related genes by sponging hsa-miR-27a-3p. Cell Death Discov. 7, 311. 10.1038/s41420-021-00709-4 34689169 PMC8542043

[B6] Chen X. Yan L. Jiang F. Lu Y. Zeng N. Yang S. (2021). Identification of a ferroptosis-related signature associated with prognosis and immune infiltration in adrenocortical carcinoma. Int. J. Endocrinol. 2021, 4654302. 10.1155/2021/4654302 34335745 PMC8318759

[B7] Cheng Y. Kou W. Zhu D. Yu X. Zhu Y. (2021). Future directions in diagnosis, prognosis and disease monitoring of adrenocortical carcinoma: Novel non-invasive biomarkers. Front. Endocrinol. 12, 811293. 10.3389/fendo.2021.811293 PMC8844185 35178030

[B8] Du R. Huang C. Liu K. Li X. Dong Z. (2021). Targeting AURKA in cancer: Molecular mechanisms and opportunities for cancer therapy. Mol. Cancer 20, 15. 10.1186/s12943-020-01305-3 33451333 PMC7809767

[B9] Faron M. Lamartina L. Hescot S. Moog S. Deschamps F. Roux C. (2022). New endpoints in adrenocortical carcinoma studies: A mini review. Endocrine 77, 419–424. 10.1007/s12020-022-03128-2 35869971

[B10] Guo J. Li W. Cheng L. Gao X. (2022). Identification and validation of hub genes with poor prognosis in hepatocellular carcinoma by integrated bioinformatical analysis. Int. J. Gen. Med. 15, 3933–3941. 10.2147/IJGM.S353708 35431572 PMC9012340

[B11] Han J. Jiang Z. Wang C. Chen X. Li R. Sun N. (2020). Inhibition of aurora-A promotes CD8(+) T-cell infiltration by mediating IL10 production in cancer cells. Mol. Cancer Res. 18, 1589–1602. 10.1158/1541-7786.MCR-19-1226 32591441

[B12] Huang C. Cheng Y. Li W. Huang Y. Luo H. Zhong C. (2022). Examining the mechanisms of huachansu injection on liver cancer through integrated bioinformatics analysis. Recent Pat. anticancer. Drug Discov. 17. 10.2174/1574892817666220511162046 35546757

[B13] Jiang X. Stockwell B. R. Conrad M. (2021). Ferroptosis: Mechanisms, biology and role in disease. Nat. Rev. Mol. Cell Biol. 22, 266–282. 10.1038/s41580-020-00324-8 33495651 PMC8142022

[B14] Lippert J. Fassnacht M. Ronchi C. L. (2022). The role of molecular profiling in adrenocortical carcinoma. Clin. Endocrinol. 97, 460–472. 10.1111/cen.14629 34750847

[B15] Liu H. J. Hu H. M. Li G. Z. Zhang Y. Wu F. Liu X. (2020). Ferroptosis-related gene signature predicts glioma cell death and glioma patient progression. Front. Cell Dev. Biol. 8, 538. 10.3389/fcell.2020.00538 32733879 PMC7363771

[B16] Long S. Zhang X. F. (2022). AURKA is a prognostic potential therapeutic target in skin cutaneous melanoma modulating the tumor microenvironment, apoptosis, and hypoxia. J. Cancer Res. Clin. Oncol. 10.1007/s00432-022-04164-1 PMC11797485 35870015

[B17] Lu H. Li L. Sun D. Duan Y. Yue K. Wu Y. (2021). Identification of novel hub genes associated with lymph node metastasis of head and neck squamous cell carcinoma by completive bioinformatics analysis. Ann. Transl. Med. 9, 1678. 10.21037/atm-21-5704 34988187 PMC8667158

[B18] Lu M. Zhou Y. Sun L. Shafi S. Ahmad N. Sun M. (2022). The molecular mechanisms of ferroptosis and its role in glioma progression and treatment. Front. Oncol. 12, 917537. 10.3389/fonc.2022.917537 36091118 PMC9450584

[B19] Luo H. Ma C. (2021). A novel ferroptosis-associated gene signature to predict prognosis in patients with uveal melanoma. Diagn. (Basel) 11, 219. 10.3390/diagnostics11020219 PMC7913108 33540700

[B20] Mete O. Erickson L. A. Juhlin C. C. de Krijger R. R. Sasano H. Volante M. (2022). Overview of the 2022 WHO classification of adrenal cortical tumors. Endocr. Pathol. 33, 155–196. 10.1007/s12022-022-09710-8 35288842 PMC8920443

[B21] Miess H. Dankworth B. Gouw A. M. Rosenfeldt M. Schmitz W. Jiang M. (2018). The glutathione redox system is essential to prevent ferroptosis caused by impaired lipid metabolism in clear cell renal cell carcinoma. Oncogene 37, 5435–5450. 10.1038/s41388-018-0315-z 29872221 PMC6173300

[B22] Minegishi S. Kinguchi S. Horita N. Namkoong H. Briasoulis A. Ishigami T. (2022). Immune checkpoint inhibitors do not increase short-term risk of hypertension in cancer patients: A systematic literature review and meta-analysis. Hypertension 79, 2611–2621. 10.1161/HYPERTENSIONAHA.122.19865 36093785

[B23] Mizdrak M. Ticinovic Kurir T. Bozic J. (2021). The role of biomarkers in adrenocortical carcinoma: A review of current evidence and future perspectives. Biomedicines 9, 174. 10.3390/biomedicines9020174 33578890 PMC7916711

[B24] Naso F. D. Boi D. Ascanelli C. Pamfil G. Lindon C. Paiardini A. (2021). Nuclear localisation of aurora-A: Its regulation and significance for aurora-A functions in cancer. Oncogene 40, 3917–3928. 10.1038/s41388-021-01766-w 33981003 PMC8195736

[B25] Ng C. K. Y. Dazert E. Boldanova T. Coto-Llerena M. Nuciforo S. Ercan C. (2022). Integrative proteogenomic characterization of hepatocellular carcinoma across etiologies and stages. Nat. Commun. 13, 2436. 10.1038/s41467-022-29960-8 35508466 PMC9068765

[B26] Peng J. Lu Y. Chen L. Qiu K. Chen F. Liu J. (2022). The prognostic value of machine learning techniques versus cox regression model for head and neck cancer. Methods 205, 123–132. 10.1016/j.ymeth.2022.07.001 35798257

[B27] Pitsava G. Maria A. G. Faucz F. R. (2022). Disorders of the adrenal cortex: Genetic and molecular aspects. Front. Endocrinol. 13, 931389. 10.3389/fendo.2022.931389 PMC9465606 36105398

[B28] Sankhe K. Prabhu A. Khan T. (2021). Design strategies, SAR, and mechanistic insight of Aurora kinase inhibitors in cancer. Chem. Biol. Drug Des. 98, 73–93. 10.1111/cbdd.13850 33934503

[B29] Shao X. Yang X. Liu Y. Song Q. Pan X. Chen W. (2022). Genetic polymorphisms in DNA repair genes and their association with risk of cervical cancer: A systematic review and meta-analysis. J. Obstet. Gynaecol. Res. 48, 2405–2418. 10.1111/jog.15325 35732591

[B30] Sun S. Zhou W. Li X. Peng F. Yan M. Zhan Y. (2021). Nuclear Aurora kinase A triggers programmed death-ligand 1-mediated immune suppression by activating MYC transcription in triple-negative breast cancer. Cancer Commun. 41, 851–866. 10.1002/cac2.12190 PMC8441052 34251762

[B31] Tang J. Yang L. Li Y. Ning X. Chaulagain A. Wang T. (2021). ARID3A promotes the development of colorectal cancer by upregulating AURKA. Carcinogenesis 42, 578–586. 10.1093/carcin/bgaa118 33165575

[B32] Tian L. Li X. Zheng H. Wang L. Qin Y. Cai J. (2022). Fisher discriminant model based on LASSO logistic regression for computed tomography imaging diagnosis of pelvic rhabdomyosarcoma in children. Sci. Rep. 12, 15631. 10.1038/s41598-022-20051-8 36115914 PMC9482627

[B33] Tsepenko V. V. Shkavrova T. G. Cherkesov V. N. Golub E. V. Mikhailova G. F. (2021). Asynchronous DNA replication of biallelically expressed genes in human peripheral blood lymphocytes as a prognostic sign of cancer. Sovrem. Tekhnologii Med. 13, 33–38. 10.17691/stm2021.13.3.04 34603753 PMC8482818

[B34] Wang F. Zhang H. Wang H. Qiu T. He B. Yang Q. (2022). Combination of AURKA inhibitor and HSP90 inhibitor to treat breast cancer with AURKA overexpression and TP53 mutations. Med. Oncol. 39, 180. 10.1007/s12032-022-01777-x 36071247

[B35] Waszut U. Taylor N. F. (2022). Use of dissected paraffin block tissue as a source of mRNA for transcriptional profiling and biomarker identification: A review, with preliminary findings in adrenocortical carcinoma tissue. Acta Biochim. Pol. 69, 273–281. 10.18388/abp.2020_5611 35623010

[B36] Wei S. Zhang J. Shi R. Yu Z. Chen X. Wang H. (2022). Identification of an integrated kinase-related prognostic gene signature associated with tumor immune microenvironment in human uterine corpus endometrial carcinoma. Front. Oncol. 12, 944000. 10.3389/fonc.2022.944000 36158685 PMC9491090

[B37] Xiao H. Xu D. Chen P. Zeng G. Wang X. Zhang X. (2018). Identification of five genes as a potential biomarker for predicting progress and prognosis in adrenocortical carcinoma. J. Cancer 9, 4484–4495. 10.7150/jca.26698 30519354 PMC6277665

[B38] Xu W. H. Wu J. Wang J. Wan F. N. Wang H. K. Cao D. L. (2019). Screening and identification of potential prognostic biomarkers in adrenocortical carcinoma. Front. Genet. 10, 821. 10.3389/fgene.2019.00821 31572440 PMC6749084

[B39] Yeoh P. Czuber-Dochan W. Aylwin S. Sturt J. (2022). Lived experience of people with adrenocortical carcinoma and associated adrenal insufficiency. Endocrinol. Diabetes Metab. 5, e341. 10.1002/edm2.341 35670031 PMC9258998

[B40] Yu S. H. Cai J. H. Chen D. L. Liao S. H. Lin Y. Z. Chung Y. T. (2021). LASSO and bioinformatics analysis in the identification of key genes for prognostic genes of gynecologic cancer. J. Pers. Med. 11, 1177. 10.3390/jpm11111177 34834529 PMC8617991

[B41] Zhang P. Chen X. Zhang L. Cao D. Chen Y. Guo Z. (2022). POLE2 facilitates the malignant phenotypes of glioblastoma through promoting AURKA-mediated stabilization of FOXM1. Cell Death Dis. 13, 61. 10.1038/s41419-021-04498-7 35039475 PMC8763902

[B42] Zheng Y. Ji Q. Xie L. Wang C. Yu C. N. Wang Y. L. (2021). Ferroptosis-related gene signature as a prognostic marker for lower-grade gliomas. J. Cell. Mol. Med. 25, 3080–3090. 10.1111/jcmm.16368 33594759 PMC7957186

[B43] Zuo C. Lv X. Liu T. Yang L. Yang Z. Yu C. (2022). Polymorphisms in ERCC4 and ERCC5 and risk of cancers: Systematic research synopsis, meta-analysis, and epidemiological evidence. Front. Oncol. 12, 951193. 10.3389/fonc.2022.951193 36033436 PMC9404303

